# Clinical update and commentary on psychiatric care for patients experiencing workplace bullying

**DOI:** 10.1177/10398562231153021

**Published:** 2023-01-20

**Authors:** Jeffrey CL Looi, Stephen Allison, Stephen R Kisely, Tarun J Bastiampillai

**Affiliations:** Academic Unit of Psychiatry and Addiction Medicine, 2219 The Australian National University, School of Medicine and Psychology, Canberra Hospital, Canberra, ACT, Australia; Consortium of Australian-Academic Psychiatrists for Independent Policy and Research Analysis (CAPIPRA), Canberra, ACT, Australia; Consortium of Australian-Academic Psychiatrists for Independent Policy and Research Analysis (CAPIPRA), Canberra, ACT, Australia; College of Medicine and Public Health, 1065 Flinders University of South Australia, Adelaide, SA, Australia; Consortium of Australian-Academic Psychiatrists for Independent Policy and Research Analysis (CAPIPRA), Canberra, ACT, Australia; School of Medicine, 1974 The University of Queensland, Princess Alexandra Hospital, Woolloongabba, Brisbane, QLD, Australia; Departments of Psychiatry, Community Health and Epidemiology, Dalhousie University, Halifax, NS, Canada; Consortium of Australian-Academic Psychiatrists for Independent Policy and Research Analysis (CAPIPRA), Canberra, ACT, Australia; College of Medicine and Public Health, 1065 Flinders University of South Australia, Adelaide, SA, Australia; Department of Psychiatry, 2541 Monash University, Clayton, VIC, Australia

**Keywords:** workplace bullying, organisational, individual, industrial advocacy, psychiatric care

## Abstract

**Objective:**

To provide a brief clinical research update and commentary advice on the practical psychiatric care of patients suffering workplace bullying.

**Conclusions:**

While there is empirical research on the prevalence and impacts of workplace bullying, there is a relative dearth of clinical research into psychiatric patient care. Accordingly, we provide commentary on practical considerations that assist in psychiatric care planning and delivery.

Workplace bullying is difficult to define scientifically. There are objective and subjective aspects that encompass actual exposure to adverse behaviour, the targeted person’s interpretation and that of other parties, including the alleged perpetrator.^1,2^ Therefore, most research has reasonably focused on self-identification, for example, *‘Bullying takes place when one or more persons systematically and over time feel that they have been subjected to negative treatment on the part of one or more persons, in a situation in which the person(s) exposed to the treatment have difficulty in defending themselves against them. It is not bullying when two equally strong opponents are in conflict with each other’.* (p. 191)^3^ The typologies of bullying broadly involve a range of psychological and social behaviours that include, but are not limited to isolation, control via abuse of working conditions, emotional abuse, discredit and denigration and devaluation of role (See following reference, esp. Table 1 in that paper).^4^

**Table 1.** Practical dos and don’ts on supporting patients experiencing workplace bullying

Do’s	Don’ts
Listen with an open mind about the patient’s experience in the workplace and ask for clarification to understand their situation	Don’t assume you understand the patient’s workplace and culture AND be wary of the potential for over-identification with the patient if the treating psychiatrist has experiences of bullying
Ask for patient’s explicit permission when their employer (including HR), industrial advocate, external healthcare assessor or rehabilitation provider before releasing information such as reports or letters	Avoid speaking to the employer (including HR), industrial advocate, external healthcare assessor or rehabilitation provider without conferring with your patient
Limit contact with patient’s legal representatives to that agreed with the patient and inform patients of any such contact	Don’t release any information without seeking permission from the patient, and medico-legal indemnity advice if needed
Inform patients of any subpoenas or legal contact from their employer or other parties	Don’t release any information without seeking permission from the patient, and medico-legal indemnity advice if needed
Recommend patient seek appropriate professional advice regarding industrial advocacy, legal matters and finances	Avoid providing advice on matters on which you are likely not expert, such as but not limited to industrial advocacy, legal matters, financial implications of leave or rehabilitation
Seek medico-legal advice from medical indemnity provider as needed to support your patient in relation to the above	Don’t go it alone – seek advice from peers in your peer review group, as supporting such patients can be challenging for a range of reasons, as above

These phenomena often occur in large hierarchical organisations where bullying is perceived within the person’s line-management structure. The reasons for self-labelling include the difficulty of researching all other viewpoints, such as a full 360-degree assessment of all involved including the alleged perpetrator and their supporters, the target and bystanders.^2^ Following pioneering Scandinavian research into the phenomenology of workplace bullying,^3,5^ interdisciplinary research has enriched the understanding of the antecedents, impacts and organisational and individual workplace-based interventions.^6,7^

The worldwide prevalence of bullying varies by definition, but the most recent meta-analysis in 2010 indicated the rate was 15%.^8^ In terms of the psychological impacts of workplace, the most recent (2015) meta-analysis (*n* = 115,783) indicated there were positive cross-sectional associations of bullying with depression (r = 0.28, CI = 0.23–0.34), anxiety (r = 0.28, CI = 0.29–0.40) and psychological stress-related conditions (r = 0.37, CI = 0.30–0.44).^9^ Pooled longitudinal studies (*n* = 54,450) showed that workplace bullying was related to mental health complaints over time.^9^ Recent Australian data found that the prevalence of workplace bullying decreased from 9.7% in 2014–15 to 8.6% in 2020–21.^10^

Despite burgeoning research, there remain knowledge shortfalls regarding causality, processes, mediators and moderators.^1^ This has hampered effective research into interventions, rehabilitation of both victims and perpetrators, as well as the remediation of work environments to prevent and treat workplace bullying.^1^ We focus on an overview of clinical interventions for victims that can be provided by psychiatrists, primarily in outpatient settings. For broader generalisability, we do not focus on patients from any particular workplace. Medico-legal psychiatric assessments and direct industrial advocacy are outside the scope of this paper. Workplace harassment, although often associated with bullying, warrants separate discussion, as there is a separate but related evidence-base and potentially, different approaches in clinical care.

## Intervention and rehabilitation for patients experiencing workplace bullying

Primary prevention aims to forestall occupational risks before they occur.^7^ Secondary prevention implies early intervention, which is practically difficult due to the insidious nature of bullying. Secondary prevention involves both investigation of complaints, as well as counselling and psychotherapy of bullying victims.^7^ Tertiary prevention refers to interventions that ensure the sustainability of treatment.^7^

There is a relative dearth of clinical psychiatric research into specialist medical care and secondary interventions for patients presenting with symptoms arising from workplace bullying. However, there is some research evidence on counselling^11^ and inpatient treatment of bullying victims.^12^ As there are specific assessment and treatment guidelines for a range of the resultant mental disorders, such as anxiety and depression, we will focus on the framing of collaborative care and practical advice on specific interventions relating to the impacts of bullying.

## Secondary prevention and treatment: General approaches

Direct workplace interventions are complex due to differing organisational and individual patient agendas. Unless employed by the work organisation, psychiatrists will not necessarily have right of entry or authority within that organisation. If psychiatrists are employed by the organisation with which the patient has a workplace bullying complaint, there is a potential material conflict of interest with the patient’s welfare. For these reasons, our focus in on the psychiatrist as a medical specialist providing a consultation service to a patient who self-labels as having been bullied in the workplace. Some additional practical tips (Do’s and Don’ts) are summarised in Table 1.

Ask the patient about the nature of the workplace, what they experienced, the person’s social network, the alleged perpetrator and their supporters, the timeline of events, sick leave utilisation and impact on productivity or career progress, as well as the resultant effects on self-esteem, anxiety, depressive and other psychiatric symptoms. Gender, race and cultural identity may be factors in why an employee is singled out for bullying, and should be explored sensitively. The role of the workplace structures, such as human resource management^13^ as well as line-management needs to be considered (See Table 2, Vignette 1.1 for an example).

**Table 2.** Clinical Vignette (This is an entirely fictionalised example)


**Vignette 1.1**
Alina, a scientist working at a government research institute, states she has been bullied by her supervising manager who initially shouted at her in full view of her subordinate staff regarding alleged underperformance. She has subsequently observed and had reported to her by co-workers, ongoing denigration by this manager regarding the allegations over the last 24 months (she has not been able to attend work for 6 months). Alina considers there is a racial and gender element in the bullying, as she is a South East Asian, and there have been derogatory comments referring to her using stereotypical Asian characterisations such as punctiliousness and formality in work interactions. She reports marked worrying, sleep disturbance, and the inability to attend work due to panic episodes. Her GP has arranged for medical leave, now up to 6 months, and has referred her to the psychiatrist for specialist advice on managing her anxiety, rehabilitation and eventual return-to-work.
**Vignette 1.2-continued**
The treating psychiatrist conducts a comprehensive assessment, covering the workplace bullying, workplace organisational responses including human resources and rehabilitation provider. Alina lodged a formal complaint about the alleged perpetrator’s behaviour 18 months ago, and states that she was then subjected to ongoing bullying by the same manager, who was allegedly exonerated by the manager’s supervisor. Her manager then launched a counter-complaint of unprofessional behaviour from Alina which remains under investigation by organisational human resources.
Alina has been advised she will be required by her public service employer to be assessed by a work-appointed psychiatrist regarding her ongoing suitability for employment. The treating psychiatrist diagnoses a generalised anxiety disorder and panic disorder recommending pharmacotherapy (SSRI) and psychological therapy, and further leave to undertake treatment. Her psychiatrist recommends that the patient seek industrial advocacy (union or legal) advice regarding the manager’s behaviour and complaint regarding the patient, as well as in relation to her work-mandated psychiatric assessment.
**Vignette 1.3-continued**
Alina has agreed to undertake a graduated return-to-work program. She indicates that she has continued to receive denigration from her supervisor in email correspondence, via phone and reported to her by work colleagues. However, her employer states that no further investigation of the bullying will occur, as her supervisor’s manager has exonerated her supervisor. In view of the ongoing bullying and harassment by her supervisor, the psychiatrist recommends that Alina undertake a graduated return-to-work program in a different work section reporting to a different supervisor. She undertakes a successful graduated return-to-work, and receiving good references from her current supervisor, begins applying for alternative employment, in view of the above experiences with her current employer. Alina transfers to another separate governmental agency in a new role as a full-time employee.
**Vignette 1.4-continued**
In her new employment, as with any workplace, Alina finds there are some difficult interactions. Due to the context of her experiences of workplace bullying, she consults her psychiatrist. Her psychiatrist explores with Alina the sense-making of the workplace bullying in how it affected her self-esteem (including racial and gender stereotyping), sensitisation to awareness of bullying, professional identity, and future career goals. Alina decides to join a science, technology, engineering and mathematics advocacy NGO as a diversity representative, and continues to explore ways to advocate for systemic change for prevention of bullying, as well as respect for diversity in her workplace. She focuses on developing new research interests and networks that will enhance her skills, opportunities and ultimately, her professional reputation, as a bulwark against future bullying. Alina also actively increases her social networks outside of work, and contact with friends and family.

The psychiatrist’s role is as a medical specialist advocate for the patient and includes the following: clarification of the workplace context, social relations and bullying; diagnosis; the need for ongoing leave; workplace and worker duty modification; psychological and pharmacotherapy; graduated return-to-work, and; if absolutely necessary medical retirement as a last resort.

Patients should be advised to seek professional industrial advocacy through the relevant union, professional organisation or legal advisor, within the legal framework that exists – in Australia this is governed by the Fair Work Act as well as Workplace Health and Safety legislation.^14^ (See Table 2, Vignette 1.2 for an example)

Sometimes, patients will make a worker’s compensation claim in relation to the psychological injury, and if the claim is accepted, introduces the compensation provider into management of rehabilitation. This adds a further level of complexity to management, as the provider may then pay the psychiatrist and other health providers for treatment of the patient. However, the psychiatrist necessarily must act in the patient’s best interests, and not as an agent for either the employer or compensation provider. For example, the worker’s compensation provider may request a report on the patient’s condition, treatment and suitability for work. The psychiatrist advises the patient of the request for information and discusses a collaborative care approach to response, based on the patient’s best interests.

When a patient suffers severe anxiety, depression and/or psychological stress, an inpatient admission may be required for severe debilitation, risk of self-harm or to facilitate a rapid change of pharmacotherapy, among other reasons. In such circumstances, patients will need private health insurance, approval from the worker’s compensation provider or an admission to hospital.

When patients are able to make a graduated return-to-work, there may be a number of workplace conditions that are recommended by the psychiatrist in the context of the bullying such as the following: changes in work duties to allow for recovery; change of supervisor away from the alleged bully and team (which may include transfer to another part of the organisation if practicable); and in some circumstances, a departure to alternative employment (See Table 2, Vignette 1.3 for an example). However, some workplaces are so toxic from a bullying and organisational cultural perspective that employees may need to leave to prevent burnout, and for their own health.^15^

Lastly, some patients have ongoing psychiatric symptoms that present an insurmountable challenge for recovery. These patients may need to consider a medical retirement, and the psychiatrist should recommend the patient seek further industrial, legal and financial advice.

Even when returning to work, and more for those unable to do so, patients face substantial existential issues from being subjected to bullying (See Table 2, Vignette 1.4 for an example). These issues can include the loss of professional identity, and the group processes of scapegoating and ostracization, as well as their impact on the patient and their family. Psychotherapy might include focus on the making sense of the bullying episode in terms of the effect on identity, how to recognise and prevent further bullying, and goals for sustainable recovery including improving professional and social supports and networks.

## Conclusions

Workplace bullying is an important clinical patient care issue for psychiatrists. We have provided a brief clinical update and commentary on clinical approaches to management.

There are a range of societal, organisational, workplace, group and individual factors related to bullying that warrant separate research, analysis of the evidence and discussion, including related workplace behaviours such as harassment, and the broader bio-psycho-socio-cultural context.
